# Attention mechanisms and emotion judgment for Korean and American emotional faces: an eye movement study

**DOI:** 10.3389/fpsyg.2023.1235238

**Published:** 2023-08-09

**Authors:** Chunghee Chung, Sungmook Choi, Hyojin Jeong, Jiyeon Lee, Hyorim Lee

**Affiliations:** ^1^School of Child Studies, Kyungpook National University, Daegu, Republic of Korea; ^2^Department of English Education, Kyungpook National University, Daegu, Republic of Korea; ^3^Department of Early Childhood Education, Keimyung College University, Daegu, Republic of Korea; ^4^Department of Home Economics Education, Kyungpook National University, Daegu, Republic of Korea

**Keywords:** facial expression, emotions, attention, emotion judgment, eye tracking

## Abstract

**Introduction:**

This study investigates attention mechanisms and the accuracy of emotion judgment among South Korean children by employing Korean and American faces in conjunction with eye-tracking technology.

**Methods:**

A total of 42 participants were individually presented with photos featuring either Korean or American children, and their task was to judge the emotions conveyed through the facial expressions in each photo. The participants’ eye movements during picture viewing were meticulously observed using an eye tracker.

**Results:**

The analysis of the emotion judgment task outcomes revealed that the accuracy scores for discerning emotions of joy, sadness, and anger in Korean emotional faces were found to be significantly higher than those for American children. Conversely, no significant difference in accuracy scores was observed for the recognition of fear emotion between Korean and American faces. Notably, the study also uncovered distinct patterns of fixation duration among children, depending on whether they were viewing Korean or American faces. These patterns predominantly manifested in the three main facial areas of interest, namely the eyes, nose, and mouth.

**Discussion:**

The observed phenomena can be best understood within the framework of the “other-race effect.” Consequently, this prototype formation leads to heightened accuracy in recognizing and interpreting emotional expressions exhibited by faces belonging to the same racial group. The present study contributes to a deeper understanding of how attention mechanisms and other-race effects impact emotion judgment among South Korean children. The utilization of eye-tracking technology enhances the validity and precision of our findings, providing valuable insights for both theoretical models of face processing and practical applications in various fields such as psychology, education, and intercultural communication.

## 1. Introduction

For human social interactions, it is important to not only know and express one’s emotions but also recognize and respond appropriately to others’ emotions. Many studies have indicated the ability to read other people’s emotions, namely emotion recognition skills, as an essential factor in interpersonal interactions (Siegman et al., 1987; Choi et al., 2014). Emotion recognition is mainly performed through verbal cues, such as speech and voice, and non-verbal cues, such as facial expressions and behaviors. According to Albert Mehrabian’s 7-38-55 communication model, words of the conversation account for 7%, tone and voice account for 38%, but facial expressions account for 55% of emotion recognition (Mehrabian, 2017). This shows that facial expressions are signals that best reflect and convey human emotional states (Ekman, 1982; Barrett et al., 2007), and they are closely tied to successful interpersonal relationships.

Recognizing faces plays a pivotal role in social interactions (Haxby et al., 2002). Surprisingly, even infants as young as 6 months old exhibit an impressive ability to differentiate between human faces and those of monkeys (Pascalis et al., 2002). However, an intriguing shift occurs around 9 months of age, where infants become increasingly skilled at distinguishing human faces specifically. This phenomenon is known as perceptual narrowing, where face perception becomes finely tuned based on individual experiences.

Further evidence of the influence of experience on face perception is reflected in the “other race effect.” Studies have shown that individuals tend to recognize and distinguish faces of their own race more accurately compared to faces of other races (Meissner and Brigham, 2001; Kelly et al., 2007, 2009). Previous studies have reported that the other-race effect is evident from the age of 9 months (Sangrigoli and De Schonen, 2004; Hayden et al., 2007; Kelly et al., 2007, 2009). A study of six-and nine-month-old infants in South Korea found that Korean infants distinguish Asian faces better than white faces, confirming that race-based selective facial recognition is a common developmental phenomenon (Kim and Song, 2011). Moreover, the other-race effect can be expected to influence the process of interacting and communicating with individuals of different races and cultures as the world enters the era of internationalization and hyper-connectedness.

Facial expressions are an important cue for emotion recognition, and the ability to recognize them begins to develop early in life (Darwin, 1872; Haviland and Lelwica, 1987; Termine and Izard, 1988). Even a 10-week-old infant can distinguish between joy, sadness, and anger in their mother’s face (Haviland and Lelwica, 1987). By the end of their first year, infants exhibit social referencing abilities, using facial expressions of others to guide their behavior in uncertain situations (Klinnert et al., 1986). Around the ages of 3–5, they can recognize basic emotions, such as joy, sadness, anger, and fear, from other people’s facial expressions (Termine and Izard, 1988; Park et al., 2007). Further, the ability to recognize complex emotions (such as embarrassment and shame) and basic emotions develops after the age of five (Yi et al., 2012). The accuracy of emotion recognition from facial expressions increases with age (Zuckerman et al., 1980; Harrigan, 1984; Markham and Adams, 1992; Nowicki and Duke, 1994), but the development of this ability varies depending on the type of emotion. For example, at 4 and 7 months old, infants display a preference for looking at happy faces compared to angry or neutral faces (Walden, 1991). And children are better at recognizing emotions of pleasure, such as joy, than emotions of displeasure, such as sadness, anger, and fear (Fabes et al., 1994). However, the development of children’s emotion recognition ability is not fully understood, especially in relation to the age at what which they are able to recognize different types of emotion.

On the other hand, it is also necessary to determine if there is a difference in emotion recognition from facial expressions due to person’s biological factors such as age, sex, and race. In particular, a difference in emotion recognition from facial expressions due to difference races is called the other-race effect, which we want to focus on in this paper.

Ekman (1972) and Izard and Malatesta (1987) verified that there are facial expressions that are universally recognized through a comparative cultural study of facial expressions. However, other studies indicate a difference in emotion recognition accuracy from facial expressions based on race (Izard, 1971; Huang et al., 2001; Elfenbein and Ambady, 2002). As such, it is difficult to conclude that facial expressions are culturally universal, which are understood in a universal way without any background based on cultural context (Gendron et al., 2014). Hence, it is necessary to conduct research on emotion recognition skills from facial expressions according to race. South Korea has historically maintained a monoculture and has been monolingual for around 5,000 years, and the pride of maintaining the population as a single race is imprinted in the Korean mindset (Kymlicka, 1996; Park, 2006). Ethnic composition of South Korea consists of 97.7% Korean, 2% Japanese, 0.1% Chinese, 0.1% U.S. white persons, and so on (Britanica, 2000). As such, international marriages and the influx of foreigners have not been active compared to other countries. Currently, most Korean children have rare opportunities to be exposed to people of other races in South Korea. Therefore, we can assume that Korean children may exhibit differences in emotion recognition from facial expressions derived from the same and other races. Moreover, as South Korea has not yet become free of multicultural challenges due to the increase in international marriages and internationalization, there is interest in the research on emotion recognition according to race and multicultural families. However, in previous research, the studied faces of other races were Southeast Asians or Japanese, similar to Koreans, or were limited to black and white persons (Ha et al., 2011; Choi et al., 2014). In addition, the participants of prior studies were elementary school students or older individuals, and there is no research on Korean children in the developmental stage of emotion recognition.

This study used eye-tracking to investigate differences in emotion recognition accuracy from facial expressions. The use of eye-tracking is based on the eye-mind link hypothesis (Just and Carpenter, 1976), which states that if we are looking at a particular object or area of a scene, it is likely that we are thinking about that object or area. Just and Carpenter (1976) found that eye fixations are closely correlated with cognitive processes, and can be used to predict the performance of participants on cognitive tasks and to identify the cognitive processes that are involved in a particular task. Eye tracking provides information about where and for how long visual attention is paid to specific stimuli (Carpenter, 1988). Furthermore, by gathering specific information on how much attention participants pay to a certain stimulus and the order in which they view it, it is possible to infer their cognitive process indirectly. Visual attention on a fixed area is the cognitive process of concentrating on information about the area (Just and Carpenter, 1980; Holmqvist et al., 2011; Lockhofen and Mulert, 2021), the eye-tracking method is considered especially useful for understanding the development of emotion recognition from facial expressions, especially among children with limited verbal expression.

Facial expressions consist of muscle movements in specific areas of the face. Therefore, to recognize emotions from facial expressions, information is collected and identified by paying attention to visual cues in areas such as the eyes, nose, and mouth. Previous studies have revealed that certain areas of the face contain more useful information for emotional identification than others, and the eyes and mouth, with the most movement making various shapes, are among the facial components that provide major cues for emotion recognition. For example, to accurately recognize emotions of joy, one has to stare at the mouth because the corners of the mouth rising serve as a decisive cue for the emotion of joy. In contrast, to accurately recognize sadness, anger, and fear, the eyes must be looked at as the gaze and size of the eye provide cues (Eisenbarth and Alpers, 2011). Individuals with schizophrenia and autism tend to be distracted and unfocused and are unable to pay attention to facial features so that they have difficulty recognizing emotions from facial expressions (Kohler et al., 2003). In particular, individuals with autism tend to stare less at the eye area, which provides an important cue for recognizing emotions (Klin et al., 2002; Spezio et al., 2007; Stephan and Caine, 2009). However, these previous studies do not imply that the nose area does not provide emotional information. There are previous studies considering nose area (Watanabe et al., 2011; Schurgin et al., 2014; Reisinger et al., 2020). In particular, nose area plays an important role on comparing pleasant and unpleasant emotions (Schurgin et al., 2014), or deaf and hearing people’s emotion recognition (Watanabe et al., 2011). While a previous eye-tracking study on facial perception of Chinese children aged 4–7 years on other-race faces found that children spend more time fixating on the nose than on other areas of the face, suggesting that the nose plays a role in facial recognition (Hu et al., 2014).

In this context, we hypothesized that Korean children would be more accurate in recognizing emotions from Korean children’s facial expression than from American children’s facial expression. We also hypothesized that Korean children would show different eye movement patterns when recognizing emotions from Korean children’s faces than from American children’s faces. Based on these hypotheses, this study addresses the following research questions: Is there a difference in the accuracy of Korean children’s emotion recognition (joy, sadness, anger, and fear) from Korean and American children’s facial expressions? Is there a difference in the eye movement patterns in Korean children’s emotion recognition (joy, sadness, anger, and fear) from Korean and American children’s facial expressions?

## 2. Materials and methods

### 2.1. Participants

The participants were six-year-old Korean children enrolled in kindergartens and daycare centers in the Daegu metropolitan area. Their participation was approved by their parents or legal guardian through a signed consent form. A total of 109 consent forms—52 from kindergarten A, 33 from kindergarten B, and 24 from kindergarten C—were collected without any restriction of children. This study was approved by the Institutional Review Board of Kyungpook National University under approval number KNU2019-0136.

The eye-tracking experiment was conducted in two phases due to the shortage of children’s concentration, which are explained below in detail. In the first phase, 46 children were deemed unsuitable for the eye-tracking experiment after calibration, who had severe myopia, astigmatism, and wore glasses, and most of them could not reach the 80% threshold in eye-tracking recognition rates. Only 52 out of the 63 children selected for the second phase actually took part. After the second phase, 10 children were excluded because they scored an eye-tracking recognition rate of less than 80%, refused to respond, or provided missing responses. The data for the final analysis consisted of data from 42 children, of which 19 were boys and 23 were girls, with an average age of 75.9 months ranging from 70 to 81 months (*SD* = 3.16).

### 2.2. Experimental tools

#### 2.2.1. Eye tracker

The eye-tracking device used in the experiment is the Tobii 1750 eye tracker (Tobii Technology, Stockholm, Sweden), which is a 17-inch-wide screen-based eye-tracking system using an IR camera with a frame rate of 50 Hz built into the bottom of the screen to track the user’s gaze. The device allows researchers to freely set areas of interest (AOIs) and extract two most commonly used eye tracking metrics by using Tobii’s ClearView analysis software such as the fixation duration for how how long the participants stare at each AOI.

#### 2.2.2. Photo stimuli

The Assessment of Children’s Emotional Skills (ACES) developed by Schultz, and the Korean ACES were used as photo stimuli by displaying them on the eye-tracking screen. The Korean ACES was modified to suit Korean domestic circumstances such as ethnic composition, by replacing the original ACES’ photos by Korean children’s photos (Chung et al., 2022). ACES’ original tools include four sets of photographs of four emotional expressions of non-Korean children including both black and white children: joy, sadness, anger, and fear. Note that only four out of six basic emotions (joy, sadness, anger, fear, disgust, and surprise) of Ekman (1999) were considered since emotions of disgust and surprise are the more complex ones such as pride, shame, and so on, and therefore they are not correctly recognized by even 5-year-old children (Shaffer, 2005).

Accordingly, photos expressing the same emotions were taken for Korean children, and a domestic validation was performed. To minimize the difference in the facial expressions of Korean and American children, children aged 5–12 attending acting institutes acted as similar as possible to the original ACES tools.

All photos were 10-inch-wide squares. Sixteen photos from the original ACES tools and another 16 photos from the Korean ACES tools were used, with each set consisting of four photos for each of the four emotions. Among these two sets of 32 photos, there are 15 girls and 17 boys, and 16 Korean, 16 American children of various races. See Supplementary Figure S1. Children sat so that their eyes were nearly two feet perpendicular to the center of the screen, which made both horizontal and vertical visual angles of approximately 23.54 degrees.

**Figure 1:**
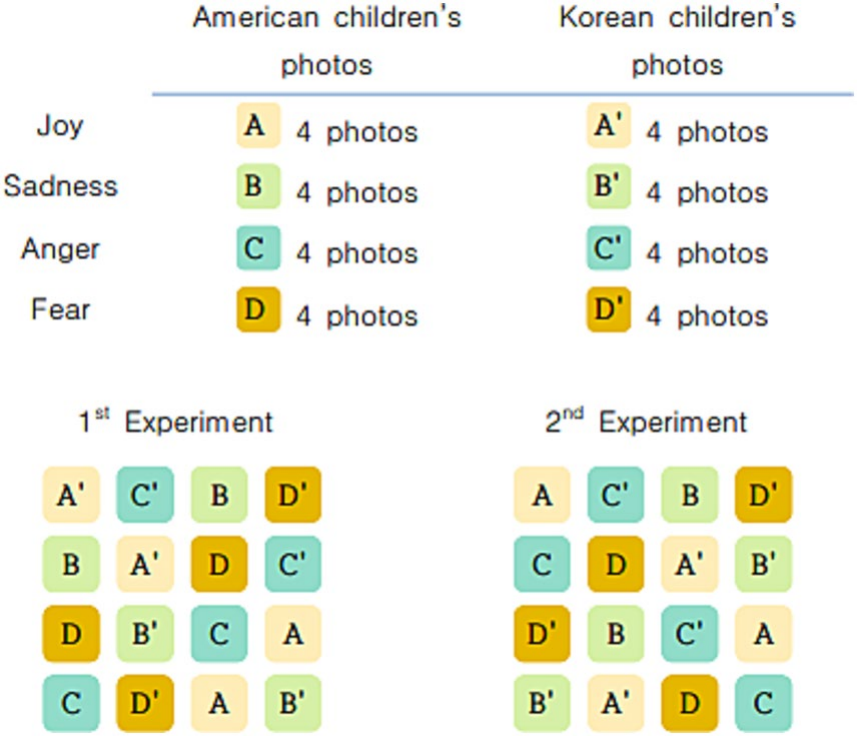
The blocking method used for photo stimuli.

Since the children’s concentration was short, the experiment was conducted in two phases, each of which shows them 16 different photos. Each set of 16 photos consisted of eight Korean and eight American children’s photos, each of which contained two photos for each emotion, and all the photos were presented using a blocking method. The blocking method entails simultaneously presenting multiple stimuli in multiple blocks so that it prevents stimuli of the same kind from appearing; for instance, photos with the same emotion or individuals of the same race were not shown more than twice in a row. In the experiment, the fixed blocking method shown in Figure 1 was used.

Two additional stimuli were used in addition to the Korean and American children’s facial photos. One was a cross-marked slide that helped focus the child’s gaze before the facial photo stimulus, and the second was a slide that was added to collect responses from the child after the facial photo stimulus was presented.

**Figure 2:**
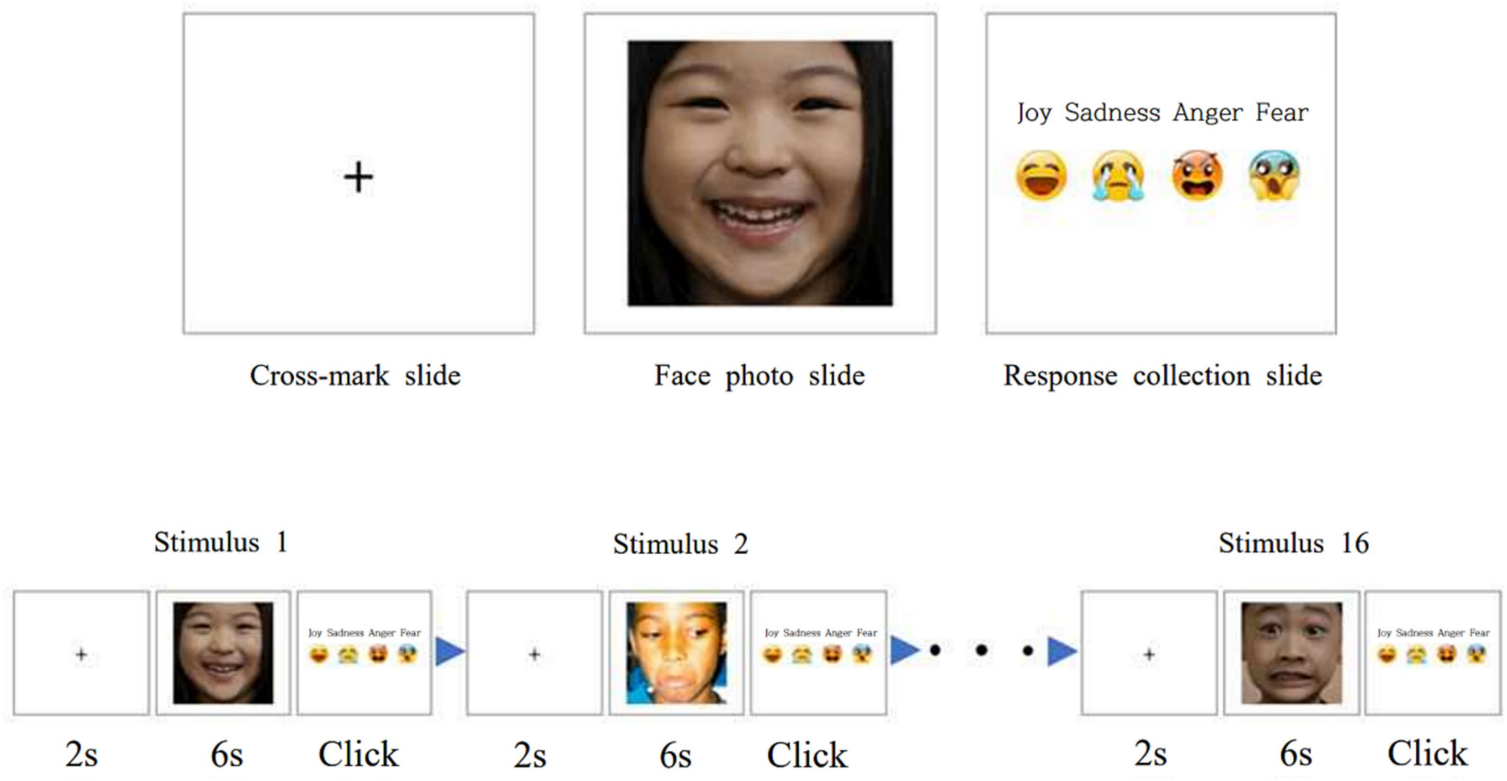
Photo stimuli Procedure. (Photograph in the stimulus 2 reproduced from ACES database with permission of David Schultz).

The cross-marked slide was presented for 2 s at first, and then, the second slide automatically appeared, with ACES measurement tools showing only children’s facial photos for 6 s. At this time, the participants were required to look at the child in the picture and infer which of the four emotions they thought was represented in the picture. They responded only when they moved on to the next slide, which was the response collection slide. The reason participants could speak only when they moved on to the response collection slide, without immediately responding to the picture, was to prevent an unnecessary gaze from being captured when they spoke.

The response collection slide, the last slide for one photo stimulus, was set up to manually move on to the next set of slides for the next photo stimulus, but only when the research assistant clicked the mouse. Then, the next three slides for the subsequent photo stimulus would be repeated. Figure 2 summarizes the process of presenting a photo stimulus and portraying it as a picture.

#### 2.2.3. Experimental procedure

Before the experiment, the researcher visited the participants’ institutions to find a suitable place for the experiment. This is because the participants could have become distracted from the eye-tracking experiment if there were external noise or stimulation. Thus, the researcher found a quiet, independent space where the participating children could remain focused. The luminance was around 300 lux on a desk, which is recommended for lighting in kindergartens or daycare centers in Korea, and it is an ideal luminance for eye tracking.

Three researchers were involved in the experiment. Researcher 1 was in charge of forming a lab with the participating children to explain and conduct the experiment. Researcher 2 was in charge of installing the eye-tracking device, adjusting it to the children’s eye level, and collecting responses. Researcher 3 was in charge of accompanying the children to the experiment site and preparing it for the next set.

First, after receiving parental consent, Researcher 3 escorted each child to the experimental site from the classroom. When the child arrived at the experimental site, Researcher 1 tried to build rapport with them so the child could adapt to the experimental environment. Researcher 1 greeted the child, talked about what they had been doing in the classroom before coming to the experiment site, and explained the experiment: “Today’s experiment involves looking at pictures of various friends on a computer screen and telling us how they feel.” Then, Researcher 1 made the child sit in the correct position. During the eye-tracking experiment, each participant was asked to keep their body as still as possible to ensure that the eye-tracking was accurate. However, children have a shorter attention span than adults, and it was difficult for them to sit still. To ensure stable data collection in cases where the participant exhibited excessive head movements, the researcher gently supported the child’s head with their hands after obtaining consent (Lee, 2019). This was done to minimize head movements and ensure the reliable acquisition of data. The authors were aware of the potential ethical issue caused by supporting children’s head, and had tried to carefully handle this issue. More precisely, we have informed about the experiment to the parents and children in detail, used a soft padded head restraint, and monitored the children closely during the experiment to ensure that they were comfortable. No participating children reported any discomfort or pain during the experiment.

**Figure 3:**
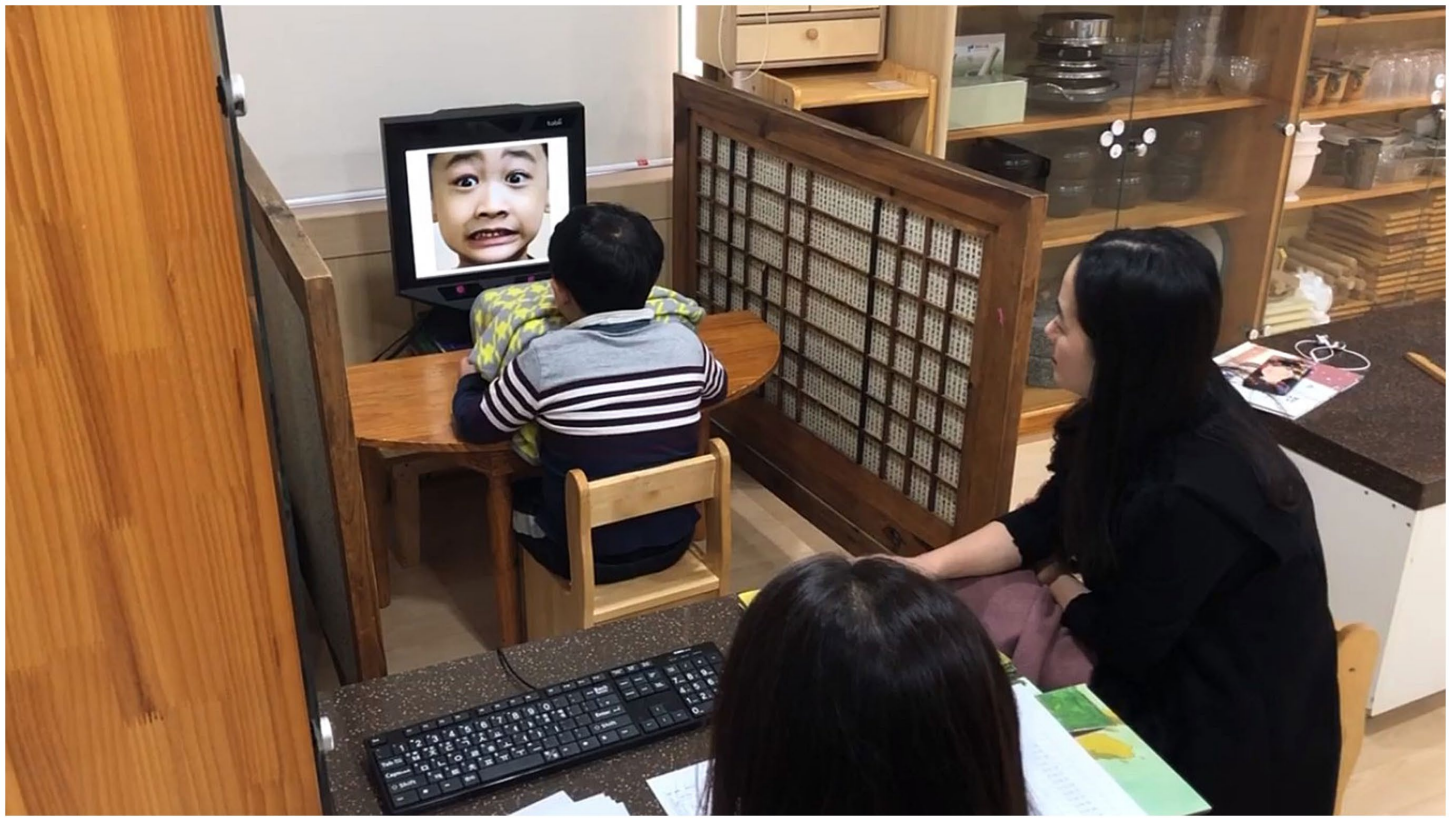
Eye-tracking experiment.

Researcher 2 helped adjust the height of the pedestal and ensured that the distance between the eye tracker and the child was appropriate based on the child’s seated height (see Figure 3).

After completing these preparations, the eye-tracking experiment began with the five-point calibration to measure whether the child’s eye movement was being properly tracked through an example stimulation. The participants practiced seeing how the photo appeared on the screen and when to respond. Subsequently, the participants were shown the 16 photo stimuli. During this process, Researcher 2 clicked the mouse button while collecting the child’s response from behind to quickly move on to the next stimulus.

The eye-tracking experiment ended after all 16 photo stimuli were shown and the responses were collected. Then, the child was checked to see if they had experienced any inconvenience during the experiment, and a return gift was given to them: a bingo game board for the first phase of the experiment and a mini block for the second phase of the experiment. The first and second phases were performed with a gap of at least 1 day. After each phase of the experiment, Researcher 3 safely dropped the child back to the classroom and brought the next child to the experiment site. It took nearly 15–20 min for one child to complete both phases of the experiment.

### 2.3. Analysis

**Figure 4:**
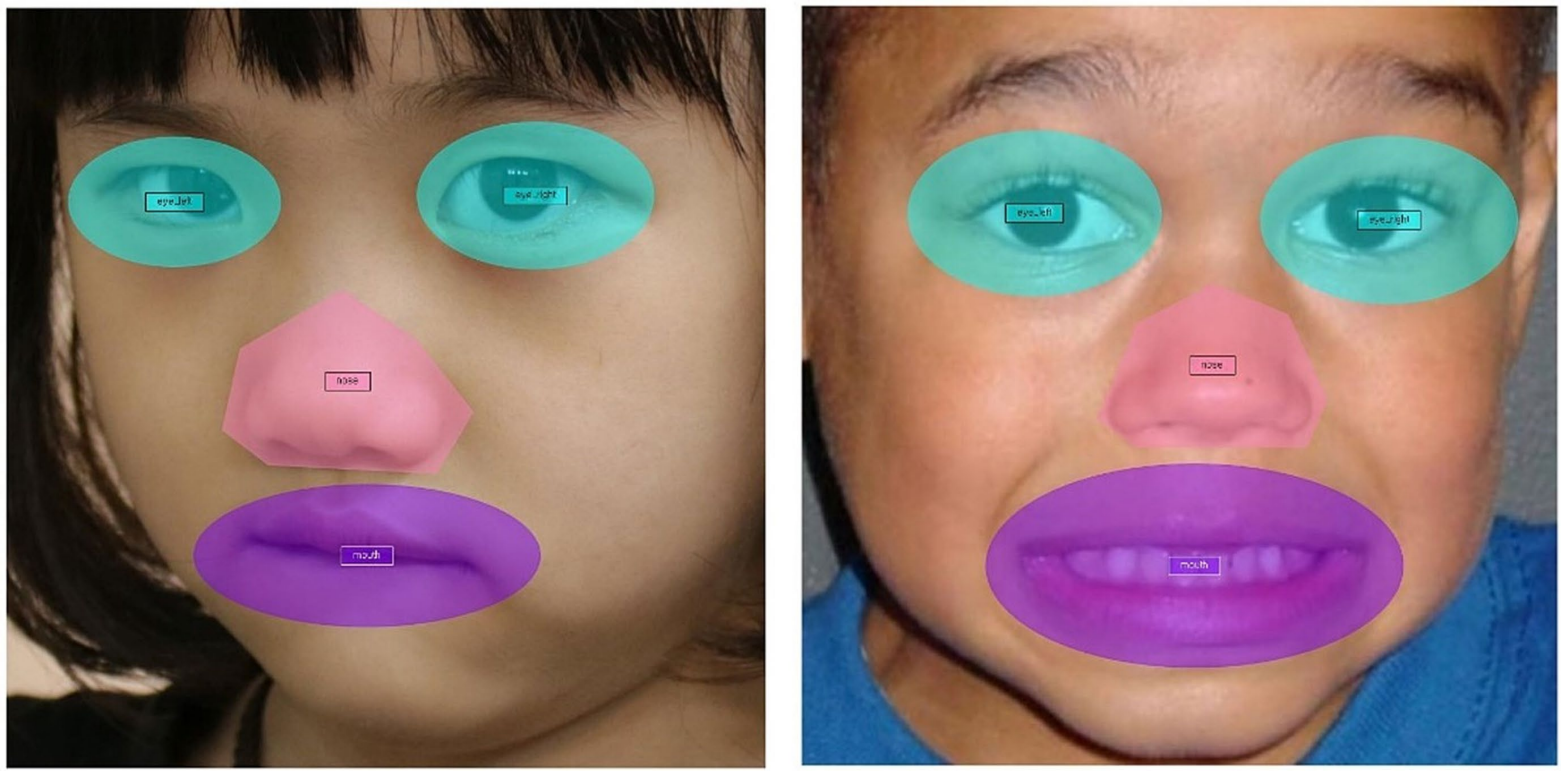
Examples of AOIs. (Photograph on the right reproduced from ACES database with permission of David Schultz).

This study tried to analyze the fixation duration where participants looked at particular facial areas to infer the emotions of the children in the pictures. The studied facial areas were the eyes, nose, and mouth. As the sizes of the eyes, nose, and mouth of the child in each photo stimulus were different, the AOIs were set individually (see Figure 4). In this study, in addition to the eyes and mouth, the nose was included as an AOI. The data extracted *via* the Tobii eye tracker involved fixation duration, which is the total time for which the eyes were fixed to each AOI.

The data were analyzed using Jamovi 1.2.27. A two-way repeated measures ANOVA (Type 3 Sums of Squares) was performed with emotion and nationality as independent variables and emotion recognition accuracy as the dependent variable. Based on the results, *post hoc* tests (Bonferroni) were conducted. Additionally, a three-way repeated measures ANOVA (Type 3 Sums of Squares) was conducted with emotion, nationality, and AOI region as independent variables, and eye fixation duration as the dependent variable. Due to significant interaction effects, separate two-way repeated measures ANOVA (Type 3 Sums of Squares) were performed for each emotion (joy, sadness, anger, fear) with AOI and nationality as independent variables, and eye fixation duration as the dependent variable. *Post hoc* tests (bonferroni) were conducted based on the results. Jamovi utilized the R package “afex” to conduct ANOVA, and it automatically selected one of the two approximations, either Kenward–Roger or Satterthwaite approximation, for estimating the degrees of freedom (*df*) and calculating *p*-values (Singmann, 2018).

## 3. Results

### 3.1. Accuracy for the recognition of facial expressions

**Figure 5:**
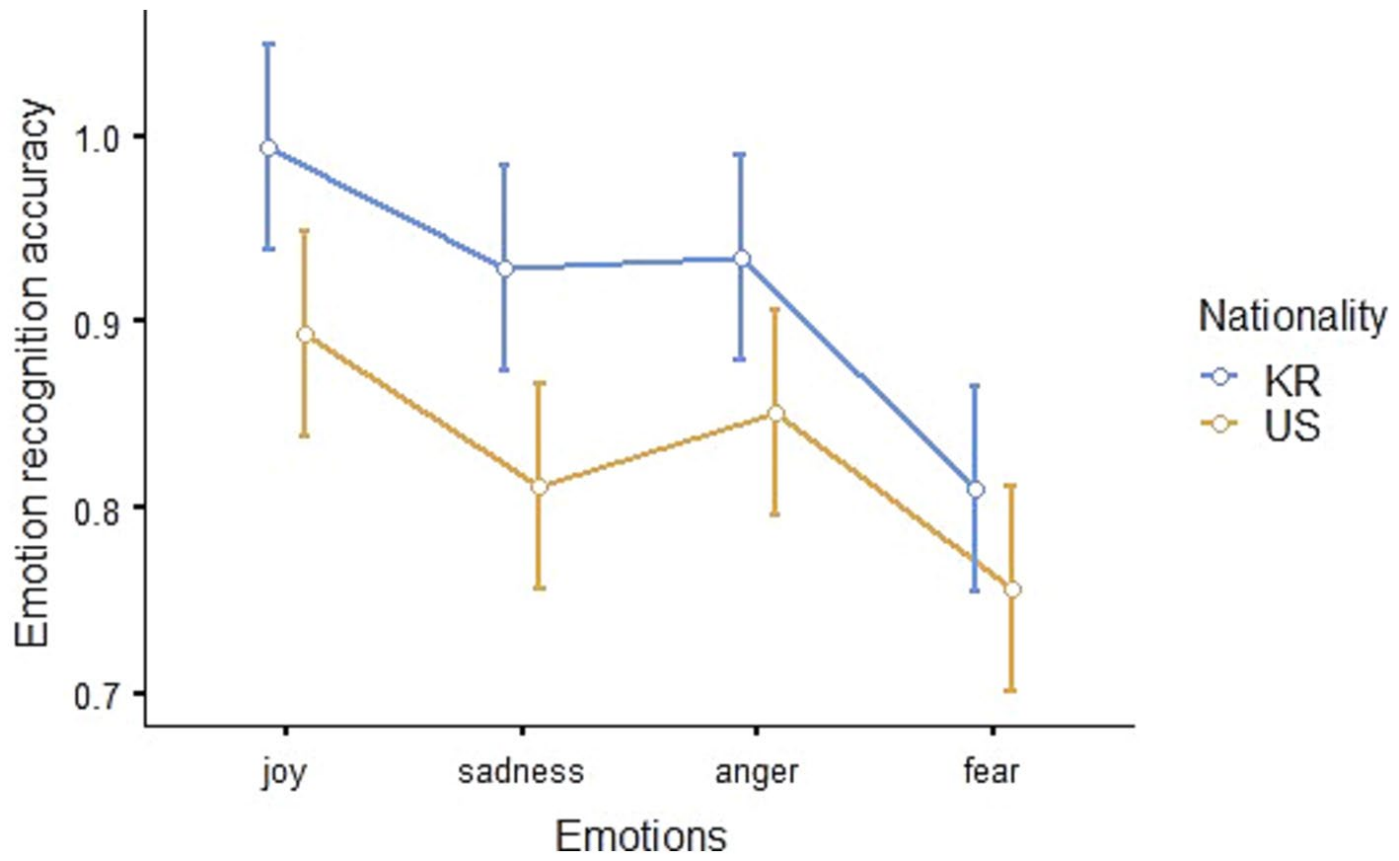
Emotion recognition accuracy according to emotion type.

The accuracy of facial recognition of Korean and American children was analyzed considering all emotion types and individual emotional types. The Estimated Marginal Means (Emotion * Nationality, *SE* = 0.028) of facial recognition accuracy is shown in Table 1, and visualized in Figure 5.

**Table 1:** Accuracy of emotional cognition in Korean and American children.

Emotion type	Korean children		American children	
	Mean	(95% CI)	Mean	(95% CI)
Joy	0.994	(0.939, 1.049)	0.893	(0.838, 0.948)
Sadness	0.929	(0.873, 0.984)	0.812	(0.756, 0.867)
Anger	0.935	(0.879, 0.990)	0.851	(0.796, 0.906)
Fear	0.810	(0.754, 0.865)	0.756	(0.701, 0.811)

A repeated measures ANOVA was performed to examine the difference in the accuracy of emotion recognition from the facial expressions of Korean and American children. There were significant differences between emotion types (*F*_3,123_ = 10.479, *p* < 0.001, *η*^2^_p_ = 0.204) and nationalities (*F*_1,41_ = 31.978, *p* < 0.001, *η*^2^_p_ = 0.438), but the interaction effect—which is the product of nationality and emotion types—was not statistically significant (*F*_3,123_ = 0.591, *p* = 0.622, *η*^2^_p_ = 0.014).

In a *post hoc* analysis by Nationality, there was also a statistically significant difference in the accuracy of emotion recognition between Korean and American children (*t*_41_ = 5.65, *p_bonferroni_* < 0.001).

In a *post hoc* analysis by emotion type, joy and fear (*t*_123_ = 5.482, *p_bonferroni_* < 0.001), sadness and fear (*t*_123_ = 2.978, *p_bonferroni_* = 0.021), and anger and fear (*t*_123_ = 3.756, *p_bonferroni_* = 0.002) were statistically significant, and the rest of the comparisons showed no significant differences.

In sum, children recognized joy, sadness, and anger more accurately than fear, and Korean children’s emotions were more accurately recognized than the emotions of American children.

### 3.2. Eye-tracking results

Fixation duration according to the type of emotion and AOI was measured *via* eye tracking. The results are depicted in Table 2. A repeated measures ANOVA was performed to examine the difference in the fixation duration to emotion recognition from the facial expressions of Korean and American children. Consequently, the interaction effect of nationality * emotion type * AOI was statistically significant (*F*_6,246_ = 14.69, *p* < 0.001, *η*^2^_p_ = 0.264). In other words, children’s fixation duration varied according to nationality and emotion type when they grasp emotions in the facial expressions of Korean and American children. Hence, for each emotion type, another repeated measures ANOVA was performed to examine the difference in fixation duration to each emotion type measured by fixation duration as well as between Korean and American children by considering the interaction effect nationality * AOI.

**Table 2:** Eye fixation duration for joy.

AOI	Korean children		American children	
	Mean	(95% CI)	Mean	(95% CI)
Eye	4.279	(3.840, 4.718)	3.806	(3.367, 4.245)
Nose	2.605	(2.166, 3.044)	4.149	(3.710, 4.588)
Mouth	4.784	(4.345, 5.223)	3.179	(2.758, 3.636)

#### 3.2.1. Eye-tracking results for joy

**Figure 6:**
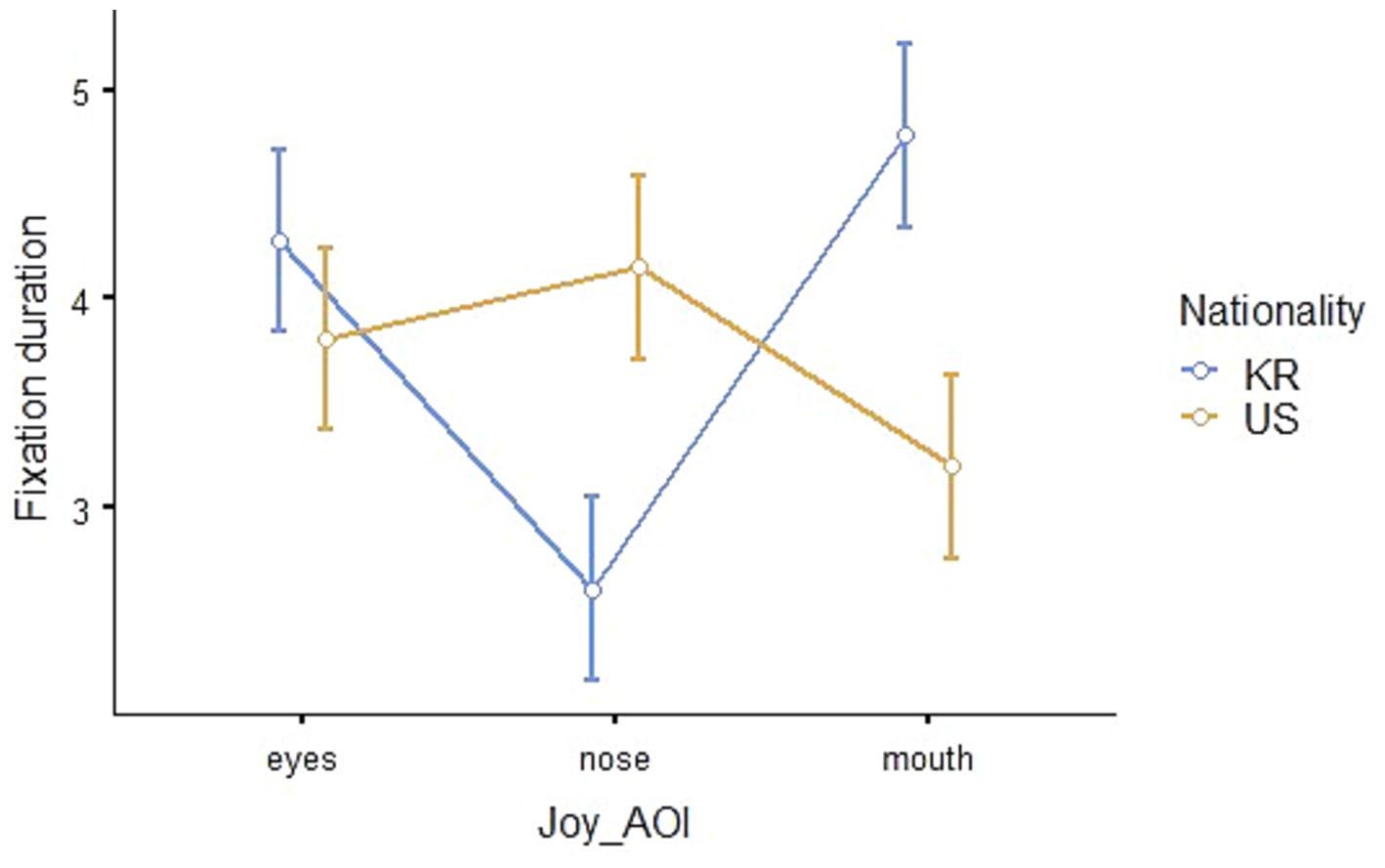
Eye-fixation duration in seconds for the emotion of joy.

The results of the repeated measures ANOVA to compare the eye-tracking outcomes for joy between Korean and American children showed that the interaction effect of nationality * AOI is statistically significant (*F*_2,82_ = 40.05, *p* < 0.001, *η*^2^_p_ = 0.494). The Estimated Marginal Means (Nationality * AOI, *SE* = 0.223) of fixation duration is shown in Table 2, and visualized in Figure 6.

The *post hoc* analysis indicated statistically significant differences between the fixation duration on the eyes and nose (*t*_136.557_ = 4.958, *p_bonferroni_* < 0.001) and the nose and mouth (*t*_136.557_ = −6.454, *p_bonferroni_* < 0.001) of Korean children. Additionally, the differences in the fixation duration on Korean children’s eyes and American children’s mouths (*t*_135.802_ = 3.215, *p_bonferroni_* = 0.024), Korean children’s noses and American children’s eyes (*t*_135.802_ = −3.571, *p_bonferroni_* = 0.007) and noses (*t*_122.960_ = −6.196, *p _bonferroni_* < 0.001) as well as Korean children’s mouths and American children’s mouths (*t*_122.960_ = 6.369, *p_bonferroni_* < 0.001) were statistically significant. Hence, the fixation duration to the eyes, nose, and mouth showed a different pattern when recognizing joy from the facial expressions of Korean and American children.

#### 3.2.2. Eye-tracking outcomes for sadness

**Figure 7:**
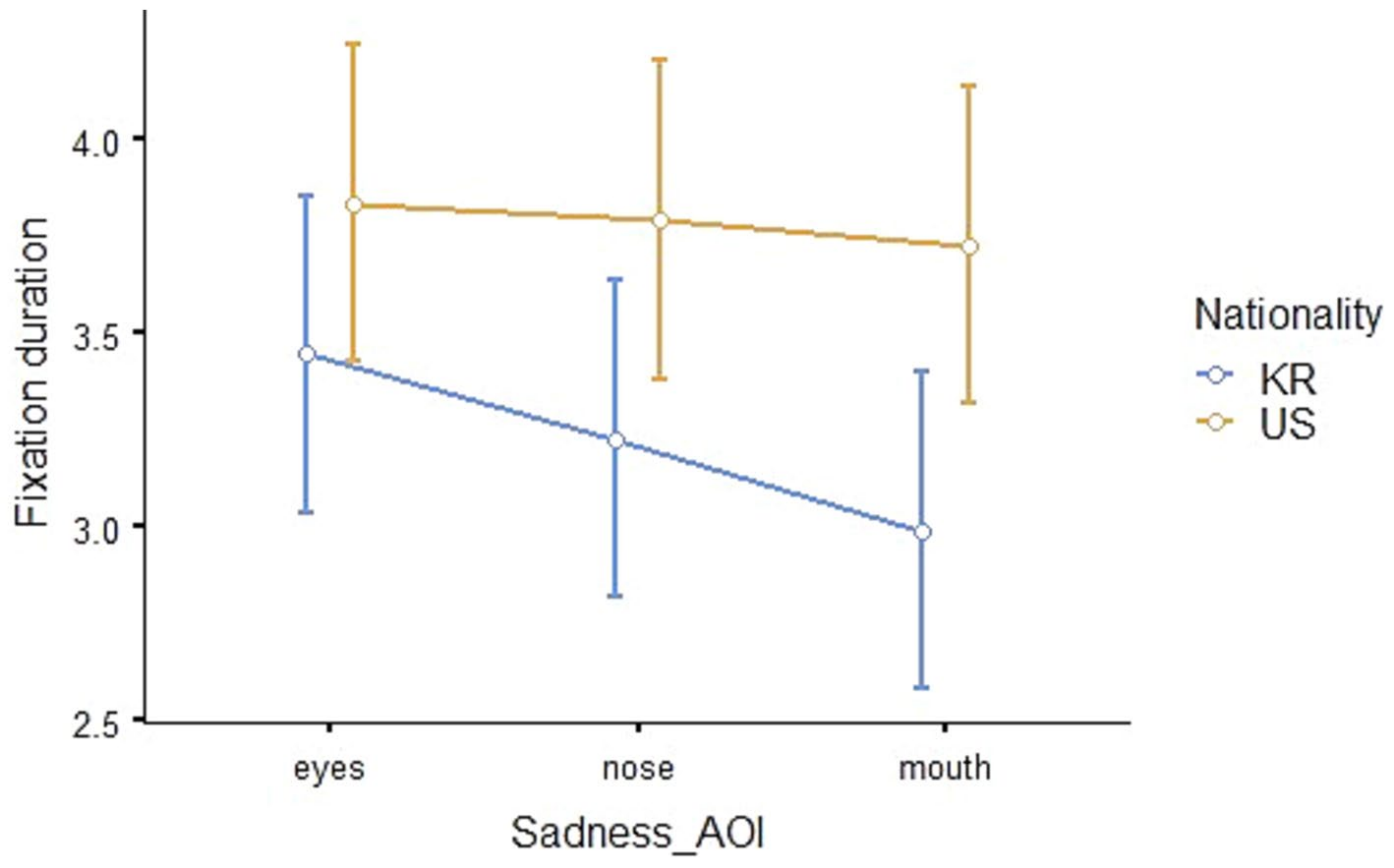
Eye-fixation duration in seconds for the emotion of sadness.

The results of a repeated measures ANOVA to compare the eye-tracking outcomes for sadness between Korean and American children revealed that the main effect of nationality was statistically significant (*F*_1,41_ = 20.187, *p* < 0.001, *η*^2^_p_ = 0.330). The Estimated Marginal Means (Nationality * AOI, *SE* = 0.208) of fixation duration is shown in Table 3, and visualized in Figure 7.

**Table 3:** Eye fixation duration for sadness.

AOI	Korean children		American children	
	Mean	(95% CI)	Mean	(95% CI)
Eye	3.446	(3.036, 3.855)	3.835	(3.425, 4.245)
Nose	3.228	(2.818, 3.638)	3.791	(3.381, 4.201)
Mouth	2.992	(2.582, 3.402)	3.727	(3.317, 4.137)

The outcome of the *post hoc* analysis indicated that the total fixation duration on the eyes, nose, and mouth of American children was longer than those of Korean children (*t*_41_ = −4.49, *p_bonferroni_* < 0.001). In other words, there was no significant difference in fixation duration on the eyes, nose, and mouth when recognizing sadness from Korean and American children’s facial expressions, but the total fixation duration on the eyes, nose, and mouths of American children was significantly longer.

#### 3.2.3. Eye-tracking results for anger

**Figure 8:**
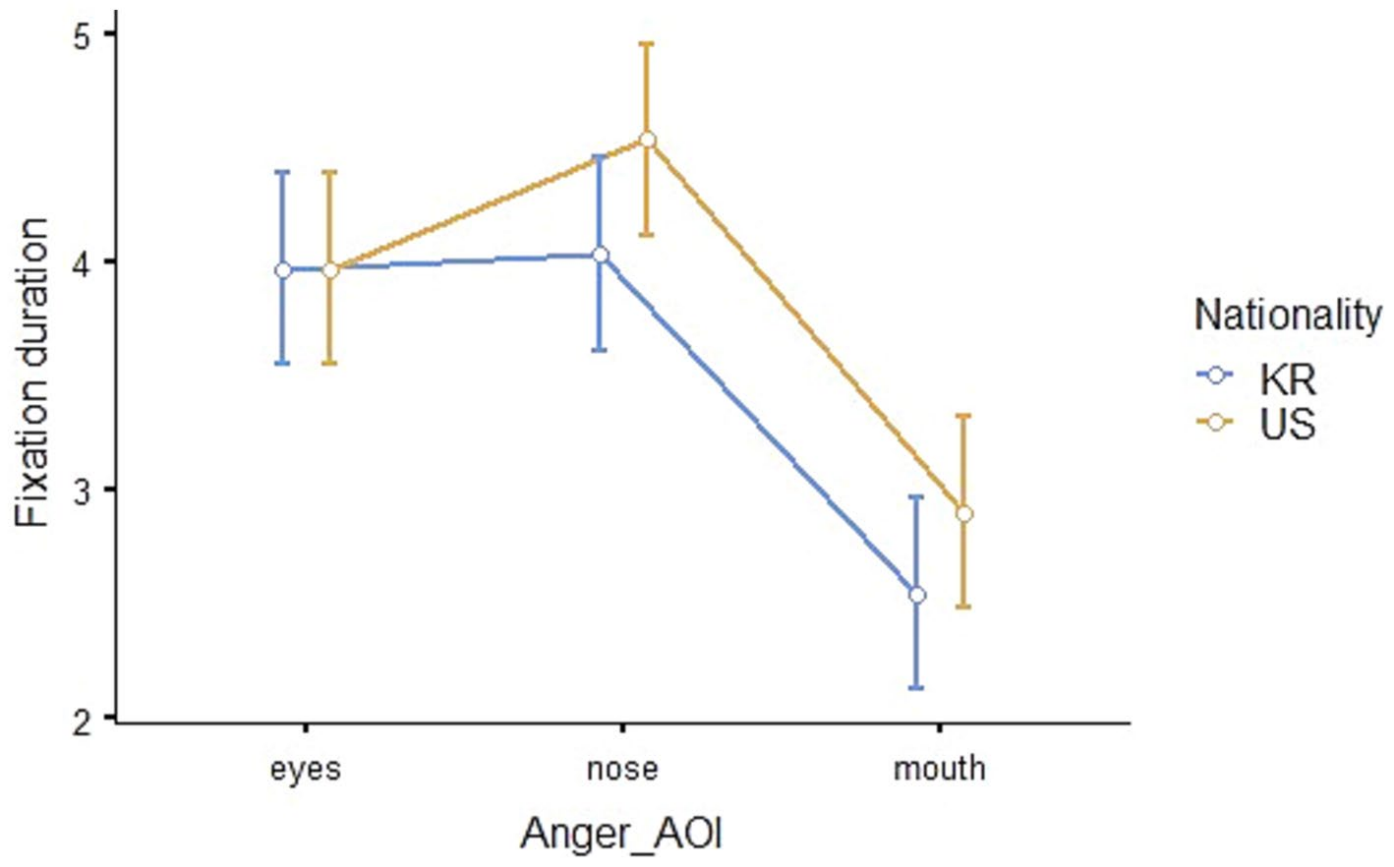
Eye-fixation duration in seconds for the emotion of anger.

The results of the repeated measures ANOVA to compare the eye-tracking outcomes for anger between Korean and American children demonstrated that the main effects of nationality (*F*_1,41_ = 9.89, *p* = 0.003, *η*^2^_p_ = 0.194) and AOI (*F*_2,82_ = 14.91, *p* < 0.001, *η*^2^_p_ = 0.267) were statistically significant, but their interaction effect was not statistically significant (*F*_2,82_ = 1.52, *p* = 0.226, *η*^2^_p_ = 0.036). The Estimated Marginal Means (Nationality * AOI, *SE* = 0.213) of fixation duration is shown in Table 4, and visualized in Figure 8.

**Table 4:** Eye fixation duration for anger.

AOI	Korean children		American children	
	Mean	(95% CI)	Mean	(95% CI)
Eye	3.967	(3.546, 4.387)	3.969	(3.548, 4.389)
Nose	4.034	(3.613, 4.454)	4.535	(4.115, 4.956)
Mouth	2.544	(2.123, 2.964)	2.899	(2.479, 3.320)

The outcomes of the *post hoc* analysis indicated that the differences in the fixation duration on the eyes and mouth (*t*_82_ = 4.12, *p_bonferroni_* < 0.001) and the nose and mouth (*t*_82_ = 5.16, *p_bonferroni_* < 0.001) were statistically significant, and the fixation duration of recognizing anger was significantly longer for American children than Korean children (*t*_41_ = −3.15, *p_bonferroni_* = 0.003).

#### 3.2.4. Eye-tracking result for fear

**Figure 9:**
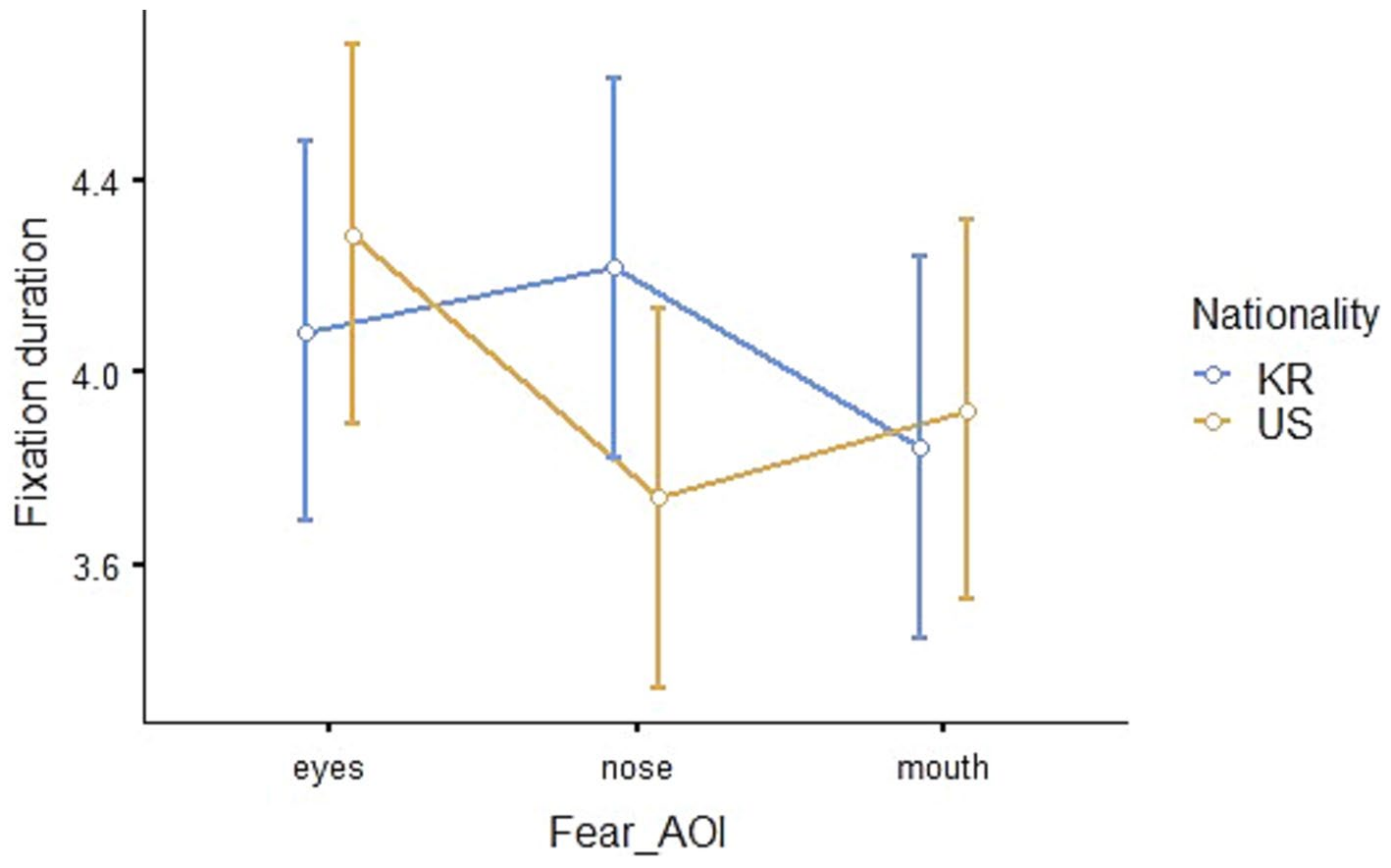
Eye-fixation duration in seconds for the emotion of fear.

The result of the repeated measures ANOVA to compare the eye-tracking results for fear between Korean and American children showed that none of the main effects of nationality (*F*_1,41_ = 0.509, *p* = 0.480, *η*^2^_p_ = 0.012) and AOI (*F*_2,82_ = 0.723, *p* = 0.488, *η*^2^_p_ = 0.017) and their interaction effect (*F*_2,82_ = 2.466, *p* = 0.091, *η*^2^_p_ = 0.057) was statistically significant. The Estimated Marginal Means (Nationality * AOI, *SE* = 0.201) of fixation duration is shown in Table 5, and visualized in Figure 9.

**Table 5:** Eye fixation duration for fear.

AOI	Korean children		American children	
	Mean	(95% CI)	Mean	(95% CI)
Eye	4.085	(3.689, 4.481)	4.287	(3.891, 4.683)
Nose	4.217	(3.821, 4.612)	3.738	(3.342, 4.134)
Mouth	3.844	(3.448, 4.240)	3.922	(3.526, 4.318)

## 4. Discussion

This study analyzed whether there was a difference between Korean and American children’s faces in emotion recognition accuracy and eye movement for 6-year-old Korean children. The suggestions derived, based on the results, are as follows. First, from examining children’s emotion recognition accuracy, the facial expression recognition accuracy of individuals of the same race was higher than that of other races for all emotion types. This result is consistent with past studies, showing a culture-based difference in emotion recognition from facial expressions (Matsumoto and Ekman, 1989; Huang et al., 2001; Elfenbein and Ambady, 2002, 2003a; Choi et al., 2014). The findings also support studies that show that the physical distance between those who express emotions and those who recognize them is related to emotion recognition accuracy (Elfenbein and Ambady, 2003b). In this regard, Hu et al. (2014) tested whether and how Chinese children aged 4 to 7 years scan faces of their own and other races differently for face recognition. In addition, this study shows that the other race effect exists not only in face recognition but also in the emotional recognition of facial expressions.

Also, it was found that children’s emotion recognition accuracy differs based on not only race but also the type of emotion. The results indicate that, regardless of race, children can recognize joy, sadness, and anger more accurate than fear. This result is consistent with previous studies that showed that some emotions can be distinguished more easily than others; specifically, pleasant emotions were found to be easier to distinguish than unpleasant ones (Field and Walden, 1982; Harrigan, 1984; Michalson and Lewis, 1985; Caron et al., 1988; Denham et al., 1990). The finding that children’s emotion recognition accuracy differs based on the type of emotion suggests that there may be different developmental trajectories for different emotions. Some emotions may be easier to recognize at younger ages, while others may take longer to develop.

Overall, the participants recognized Korean children’s facial expressions more accurately than that of American for all emotions. Thus, there was a difference in emotion recognition based on race and/or culture, indicating the influence of the other-race effect on the accuracy of emotion recognition from facial expressions. Additionally, Korean children were able to recognize joy, sadness, and anger more accurately than fear through facial expressions. This indicates that they go through the developmental process of emotion recognition in a culturally universal way. Based on the results, we concluded that children’s emotion recognition accuracy is affected by both race and emotion type.

Secondly, during the process of emotion recognition from facial expressions, children’s fixation duration exhibited different patterns on AOIs depending on whether the children were Korean and American and on the type of emotion. The patterns of children’s gaze at the eyes, nose, and mouth of Korean and American children in the pictures were different when recognizing joy. Regarding sadness, although there was a difference in whole fixation duration, the gaze patterns were similar for Korean and American children. When recognizing anger, Korean children spent more time looking at pictures of American children than pictures of Korean children, and they paid more attention to the eyes and nose than the mouth. However, no significant difference in the gaze patterns or fixation duration was observed when recognizing fear. In eye-tracking studies, the fixed visual attention of children in a particular area means that the participants are interested in that area (Goldberg and Kotval, 1999), and the fixed persistence on a certain area means that cognitive thinking about complex information is taking place in relation to that area (Henderson and Hollingworth, 1998). The findings of this study suggest that children’s visual attention patterns on AOIs are influenced by both race and emotion type. Although facial processing primarily relies on holistic strategies rather than feature-based processing, it is crucial to consider more complex fixation patterns (van Belle et al., 2010; Nakabayashi and Liu, 2014; Megreya, 2018), we may interpret the results as a difference in children’s emotion recognition of their own race and individuals of unfamiliar races.

From a developmental perspective, the medial prefrontal cortex (mPFC) appears to play a specialized role in understanding our own and others’ communicative intentions (Krueger et al., 2009; Grossmann, 2013). Even at 3 months, the prefrontal cortex is activated when babies process faces (Johnson et al., 2005). However, this response is not fully mature. In the early stages, infants’ cortical processing of faces is relatively approximate and uncoordinated, becoming more sensitive to properly positioned human faces only after further development. The brain’s left and right hemispheres are specialized for different functions, with the right hemisphere responsible for processing emotional information, particularly in facial processing. This specialization begins in the womb (Kasprian et al., 2011) and continues to develop in early life (Stephan et al., 2003). Another brain region, the superior temporal sulcus (STS), is also involved in face processing (Allison et al., 2000; Decety et al., 2013). The STS plays a crucial role in processing changing features of faces, such as eye gaze and expressions, providing essential information for social cues. Moreover, the posterior region, where the STS is located, appears to generate a configural representation of faces, essential for extracting the invariant features that make face unique. However, developmental studies have shown that the configural strategy for face processing does not fully develop until 12 years of age (Carey and Diamond, 1977).

According to Nelson (2001), as individuals age and gain experience, their ability to recognize faces becomes more specialized, leading to a narrower range of facial recognition abilities. This specialization increases the sensitivity of the face recognition system to differences in identity within one’s own species. Through extensive exposure to human faces, a mental representation of a prototype is formed, which is finely attuned to commonly observed human faces. Individual faces are then encoded based on their deviations from this prototype.

Based on this perspective, children may have different ways of recognizing emotions from facial expressions of unfamiliar race. Specifically, higher fixation duration but lower emotion recognition accuracy to American faces can be attributed to gathering more information for emotion judgment from unfamiliar faces. Even though joy is an emotion that children accurately recognize, the nationality-based difference in the gaze pattern for joy can be interpreted as a result of engagement in the emotion recognition process when handling less familiar visual data. On the other hand, Korean children looked at the entire AOI region evenly for sadness facial expressions, but they paid more attention to the eyes and nose than the mouth for anger facial expressions. Additionally, they spent more time looking at American children’s anger facial expressions compared to Korean children’s.

Lane (2000) argued that different types of emotions are represented by specific circuits that include different brain structures. The results of this study show that the differences in visual processing patterns for different types of emotions suggests that the brain has become specialized. However, emotion recognition accuracy in recognizing fear in facial expressions is not different, and the visual patterns are also not significantly different between Korean and American children’s faces. This suggests that fear is a relatively less specialized area than joy, sadness, and anger. These findings can be considered as providing empirical evidence for the eye-mind link by establishing an interpretable explanation between visual patterns and facial expression emotion recognition.

As South Korea gradually becomes a multicultural society, cultural differences in emotion recognition accuracy imply that children from multicultural families may face challenges in their peer relations and interactions. The inability to accurately recognize the emotions of children from multicultural families may negatively affect peer relationships or interactions. Hence, early childhood educational institutions need to observe whether children from multicultural families are experiencing difficulties in emotion recognition during their peer interactions. They must also provide support and create opportunities for children from multicultural families to develop and improve their ability to accurately interpret emotions from each other’s facial expressions. This can facilitate better communication and understanding among children from diverse cultural backgrounds, fostering positive relationships and interactions within a multicultural society.

### 4.1. Limitations

Since previous studies have shown that the accuracy of emotion recognition is related to language skills, social skills, and peer interactions (Chung et al., 2020), it would be meaningful to examine whether there are differences between groups through eye-tracking along with these related variables.

Although two sets of photos were chosen from the original and Korean ACES, which had been already individually validated, there was a significant difference (*t*_30_ = −5.603, *p* < 0.001) in luminance between these sets. However, the relationship between luminosity and emotion recognition accuracy turned out to be non-significant (*r* = −0.286, *p* = 0.112). We could not determine any further effect of this difference on the fixation pattern, nor could we find a previous study on the fixation pattern according to the brightness of photos. Hence it will be necessary to study changes in eye fixation pattern according to the brightness of photos.

This study was conducted on a single group of Korean children, so it is not possible to determine whether the observed effects are due to cultural or racial influences. Future studies should target children from diverse racial groups that share a common culture, such as the United States, or present photos of children from other East Asian countries to clarify the differences between race and culture.

Despite only 42 data were used for analysis while the initial sample size was 104, and so the rejection rate seems rather high. Although we could find no methodological issues, it would be better if the rejection rate could be lowered. For example, the newest eye tracker, which has better precision and accuracy, and allows a greater freedom of head movement, could be used.

Finally, the complex design of this study, which involved a three-way repeated measures ANOVA followed by post-hoc tests with Bonferroni correction, may have resulted in an overly conservative control of the Type-I error. Additionally, due to the correlated nature of the data within the two-way ANOVA framework, there is a possibility of potential false positives in the results.

## Data availability statement

The datasets presented in this study can be found in online repositories. The names of the repository/repositories and accession number(s) can be found below: https://drive.google.com/drive/folders/1o1VtUwWO0nBMdysaEjyd3IGXsRUhO1Qt?usp=drive_link.

## Ethics statement

The studies involving humans were approved by Institutional Review Board of Kyungpook National University (KNU2019-0136). The studies were conducted in accordance with the local legislation and institutional requirements. Written informed consent for participation in this study was provided by the participants’ legal guardians/next of kin. Written informed consent was obtained from the minor(s)’ legal guardian/next of kin for the publication of any potentially identifiable images or data included in this article.

## Author contributions

CC, HL, and SC contributed to the conceptualization of the study. SC and HL contributed toward the methodology of the study. HJ and JL contributed toward the investigation. SC, HL, and JL contributed toward the visualization. CC acquired the funding for the study. CC and HL were responsible for project administration and supervised the study. CC, SC, HJ, JL, and HL wrote the original draft of the manuscript. CC and HL reviewed and edited the manuscript. All authors contributed to manuscript revision, read, and approved the submitted version.

## Funding

This work was supported by the Ministry of Education of the Republic of Korea and the National Research Foundation of Korea [grant number NRF-2019S1A5A2A03051508].

## Conflict of interest

The authors declare that the research was conducted in the absence of any commercial or financial relationships that could be construed as a potential conflict of interest.

## Publisher’s note

All claims expressed in this article are solely those of the authors and do not necessarily represent those of their affiliated organizations, or those of the publisher, the editors and the reviewers. Any product that may be evaluated in this article, or claim that may be made by its manufacturer, is not guaranteed or endorsed by the publisher.

## Abbreviations

AOI: area of interest
ACES: Assessment of Children’s Emotional Skills
ANOVA: analysis of variance
