# Supplementary material for: Attention mechanisms and emotion judgment for Korean and American emotional faces: an eye movement study
Source: Front Psychol. 2023 Aug 9;14:1235238. doi: 10.3389/fpsyg.2023.1235238 (PMC10446967; doi:10.3389/fpsyg.2023.1235238)
Supplement: Supplementary file 1 [file Table_1.docx]

Supplementary Material

Attention mechanisms and emotion judgment for Korean and American emotional faces: An eye movement study

Chunghee Chung, Sungmook Choi, Hyojin Jeong, Jiyeon Lee, Hyorim Lee^*^

*** Correspondence:** Hyorim Lee: rimchild@knu.ac.kr

# Supplementary Figure

|  | Korea | | US | |
| --- | --- | --- | --- | --- |
| Joy | 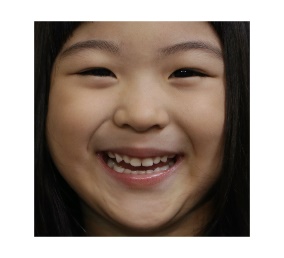 | 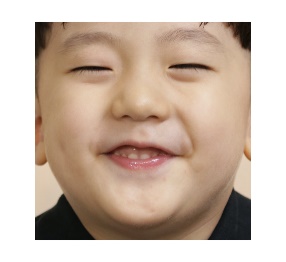 | 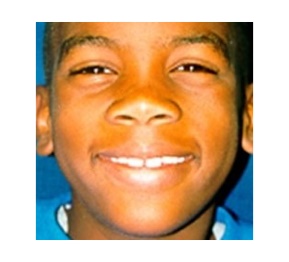 | 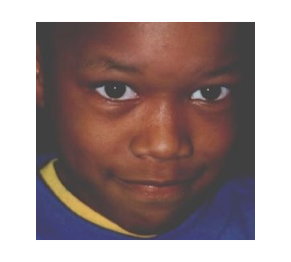 |
|  | K1 | K2 | U1 | U2 |
|  | 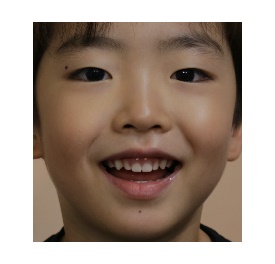 | 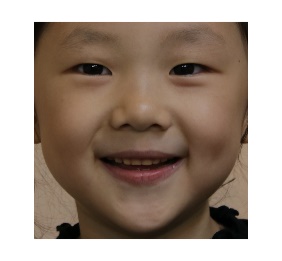 | 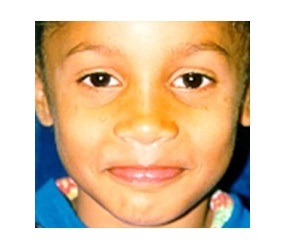 | 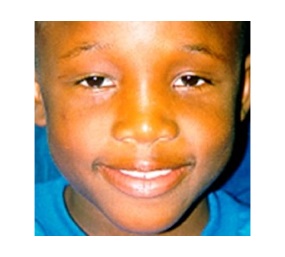 |
|  | K3 | K4 | U3 | U4 |
| Sadness | 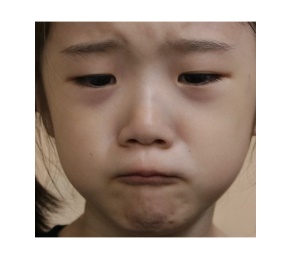 | 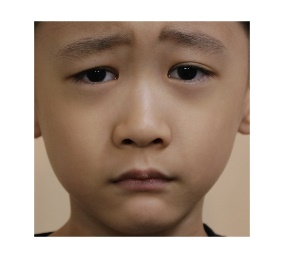 | 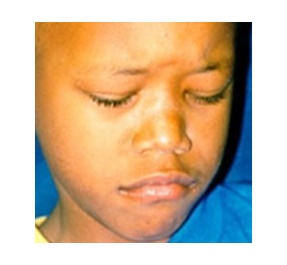 | 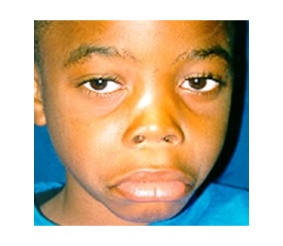 |
|  | K5 | K6 | U5 | U6 |
|  | 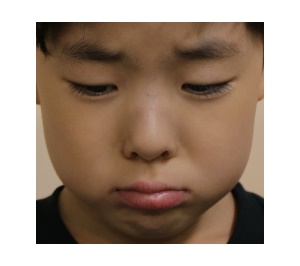 | 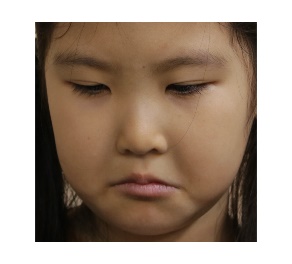 | 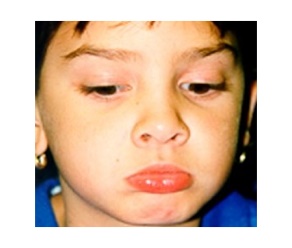 | 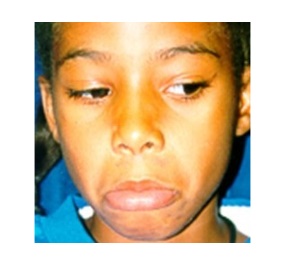 |
|  | K7 | K8 | U7 | U8 |
| Anger | 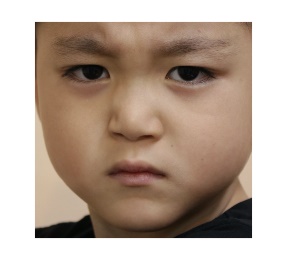 | 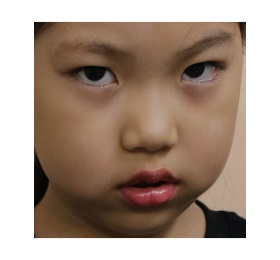 | 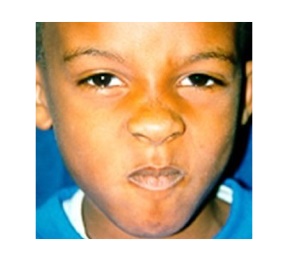 | 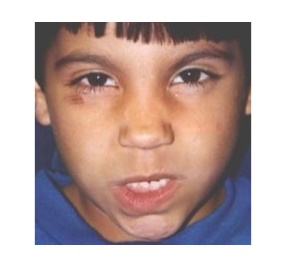 |
|  | K9 | K10 | U9 | U10 |
|  | 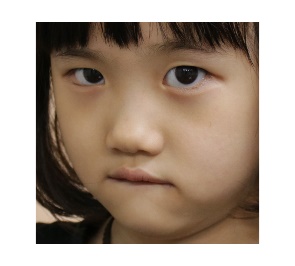 | 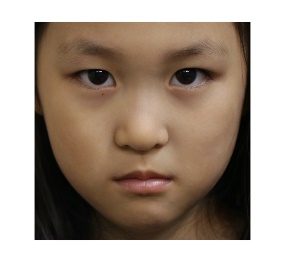 | 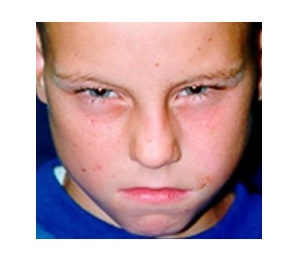 | 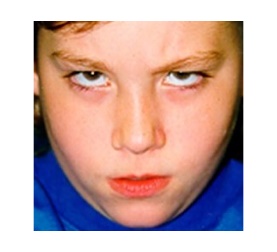 |
|  | K11 | K12 | U11 | U12 |
| Fear | 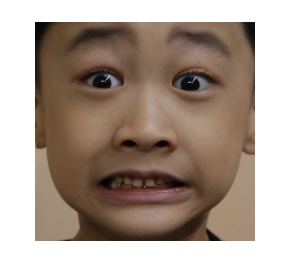 | 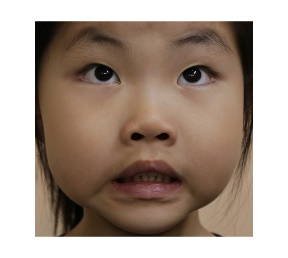 | 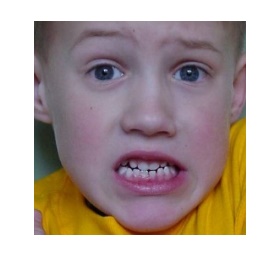 | 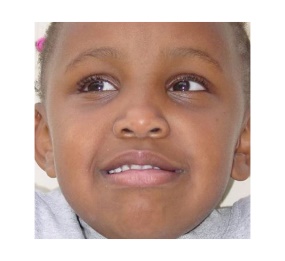 |
|  | K13 | K14 | U13 | U14 |
|  | 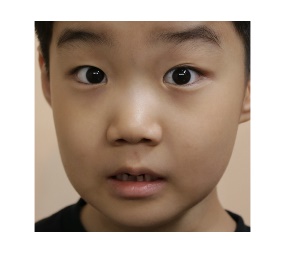 | 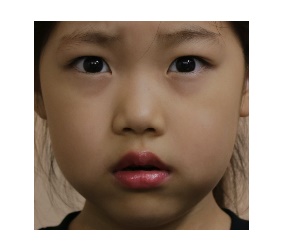 | 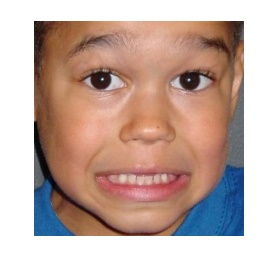 | 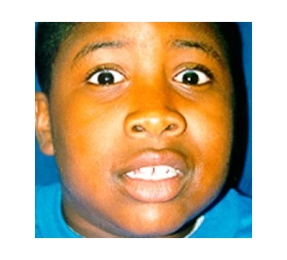 |
|  | K15 | K16 | U15 | U16 |

**Supplementary Figure 1.** The whole list of photo stimuli. The photos are presented in the following order: 1^st^ phase: K1-K9-U6-K13-U5-K2-U13-K12-U14-K5-U10-U2-U9-K14-U1-K6,

2^nd^ phase: U3-K11-U8-K15-U12-U15-K3-K7-K16-U7-K10-U4-K8-K4-U16-U11

Photograph U1~U16 reproduced from ACES database with permission of David Schultz.
